# Diaphragmatic rupture causing repeated vomiting in a combined abdominal and head injury patient: a case report and review of the literature

**DOI:** 10.1186/1749-7922-7-20

**Published:** 2012-07-02

**Authors:** Dimitrios Symeonidis, Michail Spyridakis, Georgios Koukoulis, Grigorios Christodoulidis, Ioannis Mamaloudis, Konstantinos Tepetes

**Affiliations:** 1 Department of General Surgery, University Hospital of Larissa, Mezourlo, 41110, Larissa, Greece

**Keywords:** Abdominal trauma, Diaphragmatic rupture, Multiple trauma, Motor vehicle accident, Emergency Surgery

## Abstract

**Background:**

Diaphragmatic rupture after blunt abdominal injury is a rare trauma condition. Delayed diagnosis is not uncommon especially in the emergency room setting. Associated injuries often shift diagnosis and treatment priorities towards other more life-threatening conditions.

**Case presentation:**

We present a challenging case of a young male with combined abdominal and head trauma. Repeated episodes of vomiting dominated on clinical presentation that in the presence of a deep scalp laceration and facial bruising shifted differential diagnosis towards a traumatic brain injury. However, a computed tomography scan of the brain ruled out any intracranial pathology. Finally, a more meticulous investigation with additional imaging studies confirmed the presence of diaphragmatic rupture that justified the clinical symptoms.

**Conclusions:**

The combination of diaphragmatic rupture with head injury creates a challenging trauma scenario. Increased level of suspicion is essential in order to diagnose timely diaphragmatic rupture in multiple trauma patients.

## Background

Diaphragmatic rupture (DR) after blunt abdominal trauma is a rare condition usually masked by multiple associated injuries [1,2]. The overall incidence of diaphragmatic injury is 2.5 - 5% in blunt abdominal trauma and 1.5% in blunt thoracic trauma [1]. Left sided injuries are substantially more frequent [1,2]. However, bilateral injuries have also been reported [2]. Delayed diagnosis is not uncommon especially in the emergency room (ER) setting. Despite improvement in investigative techniques a significant amount of these injuries are overlooked. Associated injuries often shift diagnosis and treatment priorities towards other more life-threatening conditions.

However, constant clinical surveillance and repeated evaluations of the patient are of paramount importance in order to minimize the likelihood of missing injuries with non-typical clinical presentation such as DR. Non-specific symptoms emanating from the respiratory system i.e. dyspnea often are the only clues for the diagnosis [3]. On the other hand, strangulation and perforation represent the final devastating consequences of the prolonged herniation of the abdominal organs into the chest [3]. Sometimes, a displaced nasogastric tube within the left hemi thorax, a diagnostic sign in chest x-ray, establishes the diagnosis of DR in asymptomatic trauma patients [3,4]. In the present report, we present a challenging case of a combined abdominal and head trauma patient. Repeated episodes of vomiting dominated on clinical presentation that on the absence of other clues shifted differential diagnosis towards a traumatic brain injury. However, a DR was finally diagnosed that justified the clinical symptoms.

## Case presentation

A 32-year-old, unrestrained male driver was involved in head-on motor vehicle accident at high speed. He was initially evaluated at the pre-hospital setting and was reported to be hemodynamically stable. On arrival, his score on the Glasgow Coma Scale was 15, blood pressure 110/75 mm Hg, pulse rate 100/min, and respiratory rate 17/min. The patent had a deep scalp laceration, signs of recent nasal bleeding and facial bruising suggestive of a high-energy head injury while he was also complaining of a mild mid-epigastrium pain.

**Figure 1:**
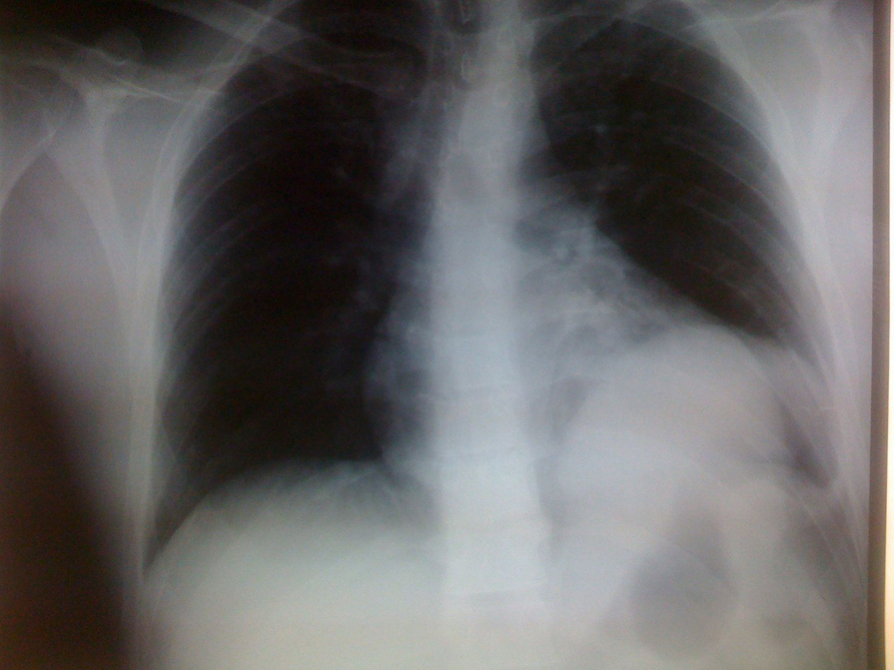
**Plain chest x-ray with the stomach in the left hemi-diaphragm**.

**Figure 2:**
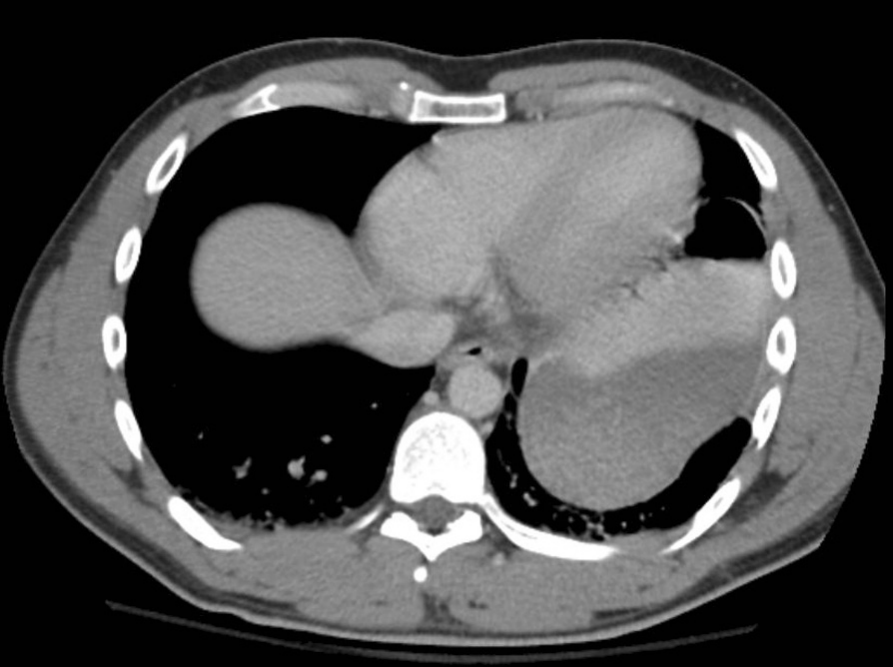
**Computed tomography scan image showing the herniation of the stomach into the chest**.

On exam, the patient was alert and oriented. The chest wall was not tender to palpation. Auscultation of the chest wall did not reveal any pathology. The abdomen was non-distended, soft with mild tenderness however to palpation of the upper abdomen (mid-epigastrium). Motor and sensory function of all extremities was intact. The urine was grossly clear. Initial radiographic studies included a supine chest film that besides a widened mediastinum was generally inconclusive. Ultrasonography in the trauma unit did not show any abnormal fluid collection. The initial hematocrit value was 39.5% and blood gas pH was 7.37 with a base deficit of 3.8. Meanwhile the patient started complaining of nausea and several blood-spotted vomiting episodes were noted. An investigation in the direction of a traumatic brain injury was conducted with a standard protocol head Computed Tomography (CT) scan. No evidences of midline shift were observed. The presence of a possible intracranial hematoma or a cranial bone fracture was ruled out. Notable oedema of the facial soft tissues, without however underlining fractures, was an additional finding. Approximately, six hours after the initial imaging evaluation, the persistence of patient’s symptoms i.e. vomiting as well as the migration of pain into the lower thorax dictated an additional workup. A second chest x-ray was obtained. (Figures 1. An elevated left hemi-diaphragm with the stomach in the left chest was observed. Abdominal CT scan confirmed the presence of a left-sided diaphragmatic tear with herniation of abdominal context within the left hemi-thorax. (Figures 2.

**Figure 3:**
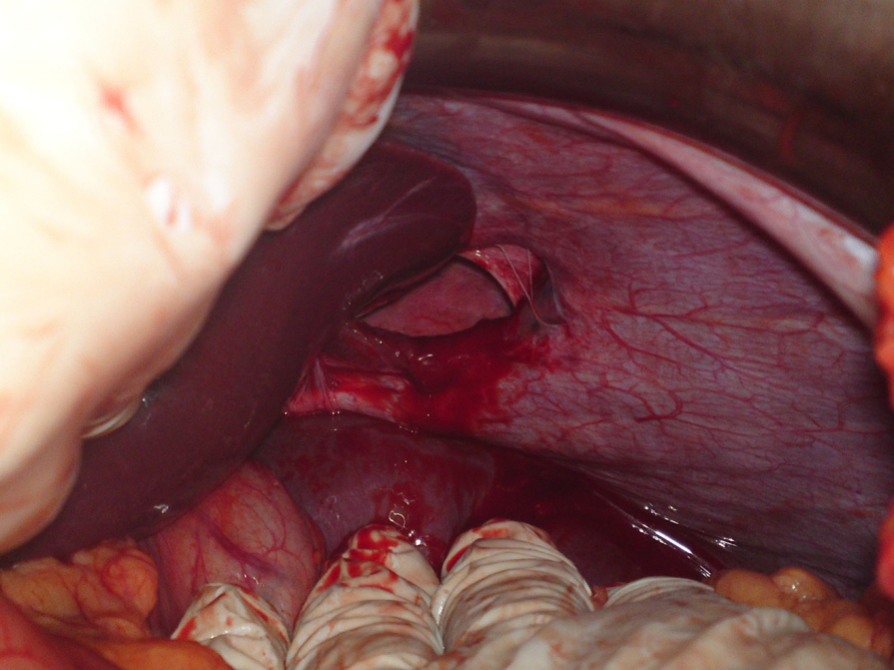
**An intraoperative photo showing the diaphragmatic defect after the reduction of the hernia contents**.

**Figure 4:**
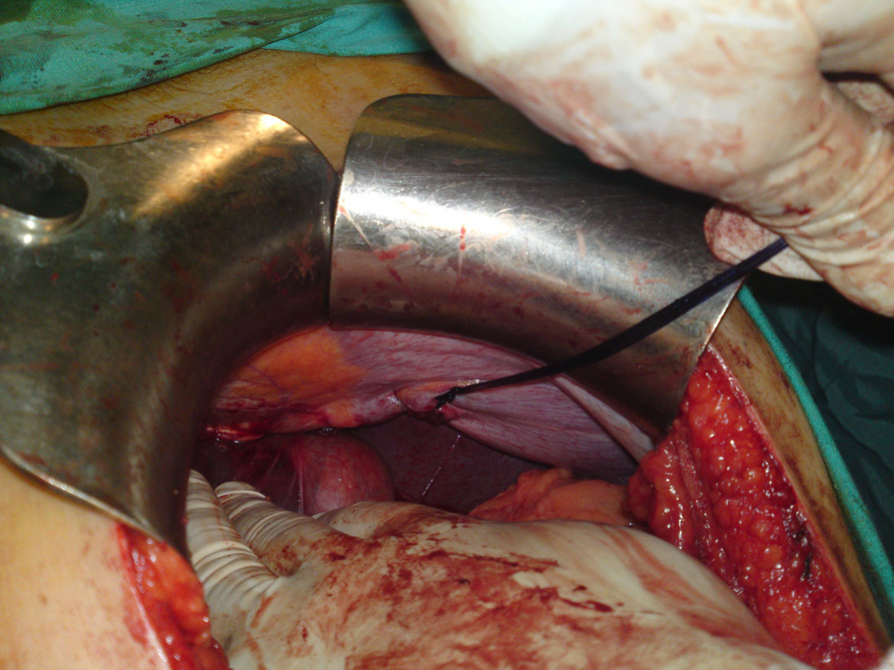
**An intraoperative photo showing the final repair result**.

The patient underwent emergency laparotomy via a midline incision where a near total herniation of the stomach into the left hemithorax was observed. No resection was necessary as there were no ischemic changes or signs of perforation of the involved organ. The stomach was then successfully reduced into the abdomen revealing the hernia opening about 5 cm in length. (Figures 3. A primary repair with interrupted non-absorbable sutures was carried out without the use of a prosthetic mesh. (Figures 4. The relatively small size of the hernia opening was the main argument for this approach. A chest tube was not necessary as pleura was not violated and a pneumothorax was not present. Operating time was 45 minutes. The patient had an uneventful postoperative period and was discharged on the fifth postoperative day.

## Discussion

DR after blunt abdominal injury is a rare trauma condition. Correct diagnosis is often difficult and is usually established late raising significantly the associated mortality and morbidity. Single or serial plain chest radiographs with a high index of suspicion are diagnostic in many cases of DR [1,4,5]. However, missed cases result in herniation of the abdominal organs into the chest which finally enlarges the diaphragm defect. Chronic intermittent abdominal or chest pain, constipation, strangulation and perforation of the involved abdominal viscera are symptoms and consequences associated with the progressive herniation of the abdominal organs into the chest. As lung on the affected side is compressed, shortness of breath, dyspnea, and respiratory infections appear [3].

Tears of the diaphragm usually originate at the musculotendinous junction, mostly in the posterolateral aspect of the hemidiaphragms. The majority of these tears are on the left side. Either the relative weakness of the left hemidiaphragm or the protective effect of the liver on the right side represents possible explanations. Irrespective of the cause, right-sided rupture is associated with increased severity of injury and, therefore, increased mortality and morbidity rates [6]. Approximately 80-90% of diaphragm injuries are related to automobile accidents. Falls or crush injuries to the diaphragm are rarer injury mechanisms. Lateral-impact automobile accident is three times more likely to cause a DR than any other impact type [7,8].

The usual scenario is the combination of DR with other types of injuries. Thoracic aortic tears, rib fractures, splenic injuries, pelvic fractures and hepatic injuries are the commonest associations [9]. Although this appears more as an observation with limited responsiveness in clinical practice, it could collectively identify patients at risk for blunt diaphragmatic rupture when certain injury patterns show up. A more expeditious and thorough work up in the right direction, i.e. diaphragmatic trauma is the minimum benefit for the multiple trauma patient [9]. On the other hand, head injuries, regardless of the severity, are not usually associated with concurrent blunt DR. Wide variations in the incidence of this injury combination are the rule in the literature. Table 1. Single institutions experience with remarkable variations in diagnostic and treatment tactics expressed via relatively small case series represent the vast majority of the reported cases. However, despite the relatively limited correlation between these two conditions - DR and head injury - complications due to a concurrent head injury accounted for the majority of deaths in a series of sixty patients with blunt abdominal trauma and DR [10].

**Table 1:** Representative case series with combined diaphragmatic rupture (DR) and head injury

	**Total number of patient with DR**	**Combined DR and head injury patients**	**% Co - existence**
Simpson et al. 2000 [11]	16	4	**25,0%**
Chen et al. 1991 [12]	62	3	**4,8%**
Pfannschmidt et al. 1994 [13]	58	22	**37,9%**
Balci et al. 2004 [14]	137	33	**24,0%**
Ilgenfritz et al. 1992 [15]	52	21	**40,3%**

As soon as the diagnosis of a DR is established a surgical repair is warrant to prevent possible complications. A midline laparotomy is the advocated approach for repair of acute diaphragmatic trauma as it offers the possibility of diagnosing and repairing other associated intra-abdominal injuries. However thoracoscopy or laparoscopy in hemodynamically stable patients represents valid alternatives for the diagnosis and repair of a missed diaphragmatic injury especially in cases of penetrating left thoraco-abdominal trauma. Generally, repair with non-absorbable simple sutures is adequate in most cases [16]. The use of mesh should be reserved for chronic and large defects [16,17].

In our case, the combined abdominal and head injury confused the diagnostic field. Concomitant maxillofacial injuries as well as the deep scalp laceration in a patient with repeated episodes of vomiting rendered traumatic brain injury as the most likely diagnosis. However, the imaging investigation ruled out a central nervous system lesion as the cause of the patient’s symptoms i.e. vomiting. The consistency of symptoms as well as the alterations of pain characteristics during the initial phase of patient’s observation was the main arguments for the additional imaging workup [18]. The pathognomonic sign in the chest x-ray with the stomach or the nasogastric tube in the hemithorax was not present in the chest radiography conducted at the trauma resuscitation unit. However, a nasogastric tube placement was contraindicated in our patient due to maxillofacial injuries and additionally a high quality chest x-ray could not be obtained until a work-up that could reliably rule out a cervical spine injury conducted.

Within the framework of a more meticulous investigation in order to delineate occult pathology to justify the clinical symptoms, a second chest x-ray under more appropriate conditions at the radiology department was obtained. The presence of the stomach within the left hemithorax was observed. Abdominal CT scan confirmed the herniation of the stomach into the chest and additionally ruled out any associated intraabdominal injuries. An urgent laparotomy at the base of DR was conducted. Regarding the repair technique we used intermittent non absorbable suture material in order to approximate the edges of the diaphragmatic defect. We assumed that the use of a prosthetic mesh in the given case with the relatively small diaphragmatic defect would increase the risk of infection and the procedure cost without corresponding benefits in the long term.

## Conclusions

Increased level of suspicion is essential in order to diagnose timely blunt DR in multiple trauma patients. Early diagnosis can lead to the proper surgical management and reduce the incidence of hernia related complications.

## Consent

Written informed consent was obtained from the patient for publication of this Case report and any accompanying images. A copy of the written consent is available for review by the Editor-in-Chief of this journal.

## Competing interests

The authors declare that they have no competing interests.

## Authors’ contribution

SD, CG, KG and MI acquired the data and drafted the article. SD, SM and TK analysed and interpreted the data. SD and TK critically revised the article. SM, CG, SD and KG performed the surgical operation. All authors read and approved the final manuscript.
